# Categorising ultra-processed foods in large-scale cohort studies: evidence from the Nurses’ Health Studies, the Health Professionals Follow-up Study, and the Growing Up Today Study

**DOI:** 10.1017/jns.2021.72

**Published:** 2021-09-16

**Authors:** Neha Khandpur, Sinara Rossato, Jean-Philippe Drouin-Chartier, Mengxi Du, Euridice M. Steele, Laura Sampson, Carlos Monteiro, Fang F. Zhang, Walter Willett, Teresa T. Fung, Qi Sun

**Affiliations:** 1 Department of Nutrition, School of Public Health, University of São Paulo, Av. Dr. Arnaldo, 715, São Paulo, Brazil; 2 Department of Nutrition, Harvard T.H. Chan School of Public Health, Boston, MA, USA; 3 Department of Public Health, Federal University of Uberlandia, Uberlandia, Minas Gerais, Brazil; 4 Center for Nutrition, Health and Society (NUTRISS), Institute for Nutrition and Functional Foods (INAF), Laval University, Quebec, QC, Canada; 5 Faculty of Pharmacy, Laval University, Quebec, QC, Canada; 6 Department of Public Health & Community Medicine, Friedman School of Nutrition, Tufts University, Medford, MA, USA; 7 Department of Epidemiology, Harvard T.H. Chan School of Public Health, Boston, MA, USA; 8 Channing Division of Network Medicine, Department of Medicine, Brigham and Women's Hospital and Harvard Medical School, Boston, MA, USA; 9 Department of Nutrition, Simmons University, Boston, MA, USA

**Keywords:** Cohort studies, Diet categorisation, Expert discussion, Food frequency questionnaires, Nova groups, Ultra-processed foods, FFQ, food frequency questionnaire, GUTS, Growing Up Today Study, NHS, Nurses’ Health Study

## Abstract

This manuscript details the strategy employed for categorising food items based on their processing levels into the four NOVA groups. Semi-quantitative food frequency questionnaires (FFQs) from the Nurses’ Health Studies (NHS) I and II, the Health Professionals Follow-up Study (HPFS) and the Growing Up Today Studies (GUTS) I and II cohorts were used. The four-stage approach included: (i) the creation of a complete food list from the FFQs; (ii) assignment of food items to a NOVA group by three researchers; (iii) checking for consensus in categorisation and shortlisting discordant food items; (iv) discussions with experts and use of additional resources (research dieticians, cohort-specific documents, online grocery store scans) to guide the final categorisation of the short-listed items. At stage 1, 205 and 315 food items were compiled from the NHS and HPFS, and the GUTS FFQs, respectively. Over 70 % of food items from all cohorts were assigned to a NOVA group after stage 2. The remainder were shortlisted for further discussion (stage 3). After two rounds of reviews at stage 4, 95⋅6 % of food items (NHS + HPFS) and 90⋅7 % items (GUTS) were categorised. The remaining products were assigned to a non-ultra-processed food group (primary categorisation) and flagged for sensitivity analyses at which point they would be categorised as ultra-processed. Of all items in the food lists, 36⋅1 % in the NHS and HPFS cohorts and 43⋅5 % in the GUTS cohorts were identified as ultra-processed. Future work is needed to validate this approach. Documentation and discussions of alternative approaches for categorisation are encouraged.

## Introduction

Ultra-processed foods are ready-to-eat/heat industrial formulations of food substances that have been derived from whole foods, and that typically contain added flavours, colours and other cosmetic additives^(1)^. They are one of the four groups that make up the NOVA classification – a system that classifies food based on the extent and purpose of the industrial processing they undergo and accounts for the physical, biological and chemical methods used in their manufacture, including the use of additives^(1)^. Recently, observational studies have provided the first evidence for the health harms associated with the intake of ultra-processed foods^(2–4)^.

For the most part, the large-scale prospective cohort studies that have assessed the associations between ultra-processed foods and disease outcomes have used a self-administered food frequency questionnaire (FFQ) for repeated dietary assessment^(5–9)^. However, the food lists on the basis of which dietary information is collected are designed to have a limited number of pre-defined items which represent the primary sources of energy and nutrients in the population under study^(10)^. As such, FFQs are unable to cover the full spectrum of foods consumed, including ultra-processed foods. Additionally, all supporting information that would be useful in identifying the ultra-processed products from the food lists like cooking methods used, food combinations and ingredients, place of food consumption and brand names of packaged products are usually not captured by FFQs.

Capturing ultra-processed food intake may not have been the explicit goal of epidemiologic studies at the time of their inception. This would have implications for the development of their dietary assessment instruments. Even 24-h diet recalls or diet records that describe with some detail the foods eaten and their method of preparation, may therefore still lack the granularity needed to accurately identify all ultra-processed products. This may result in some ambiguity in the identification of the grade of processing of a subset of food items from different dietary assessment methods and not limited to FFQs, creating an opportunity for discussion of the possible approaches for improving the identification and estimation of ultra-processed foods in epidemiologic studies.

There is limited documentation of the approaches used in the classification of food intake into the four NOVA categories or in the identification of ultra-processed foods. To our knowledge, no previous study has explicitly presented the process used in the identification of ultra-processed foods from FFQs in sufficient detail to aid replication. As a result, the challenges encountered in the process of manual classification of the diet, or the decisions made to address them have not been systematically documented. Potential sources of misclassification may also be overlooked. This gap in the evidence base hinders progress in streamlining the application of the NOVA classification to dietary intake, in identifying ultra-processed foods, in estimating their contribution to the diet, and in improving dietary assessment methods to better capture food processing levels.

The purpose of this manuscript is to detail the approach for categorising food items captured by semi-quantitative FFQs of the Nurses’ Health Studies I and II, the Health Professionals Follow-up Study (HPFS) and the Growing Up Today Study I and II, into the four NOVA groups and to identify ultra-processed foods. Collectively, these cohorts have made important contributions to the evidence base informing dietary guidelines and nutrition policy^(11)^ and their semi-quantitative FFQs have served as a template for FFQs used in epidemiologic studies, globally^(12–14)^. Presenting the strategy adopted in these cohorts will inform the categorisation of the diet in other studies that use similar assessment methods. While no validation work is presented, the broader goal of this manuscript is to encourage discussions on approaches to categorise dietary data into the NOVA groups and to inform the evolution of dietary assessment methods.

## Methods

### Cohort details

The first cohort of the Nurses’ Health Study (NHS) in 1976 enrolled 121 701 female registered nurses, aged 30 and 55 years^(15)^. In 1986, 51 529 male health professionals, aged 40 and 75 years, comprised the first cohort of the HPFS^(16)^, and in 1989, the first cohort of the NHS-II began with 116 686 female registered nurses, aged 25–42 years. Since then, participants in all three adult cohorts complete a biennial follow-up questionnaire on their medical history, lifestyle factors and occurrence of chronic diseases.

In 1996, the children of the NHS-II study participants were recruited into a study of their own – the Growing Up Today Study (GUTS)^(17)^. At inception, GUTS included 16 882 girls and boys aged between 9 and 14 years. In 2004, the study expanded to include a second cohort of 10 920 children between the ages of 10 and 17 years – the GUTS-II cohort. The two youth cohorts were followed biannually until 2013 when they were merged into one cohort.

### Assessment of food intake

Dietary data from all waves of both the adult and the youth cohorts were collected using a self-administered, semi-quantitative FFQ^(15,17)^. The first FFQ used in 1984 had 116 items and information on the usual intake of food and beverages from the NHS participants. This FFQ was expanded in 1986 to ~130 foods and sent every 4 years to track the diets of participants in both the NHS and the HPFS cohorts (NHS-II, since 1991). The GUTS FFQ included ~150 food items and was modified from the validated adult FFQ to the cognitive level and dietary knowledge of adolescents. It specifically included snack foods consumed by a younger population and food eaten away from home^(17)^. The FFQs continue to be updated to capture more detailed information on cooking methods and relevant food items^(15)^.

All FFQs ask participants how often, on average, they consumed a given reference portion of a food item over the course of the previous year. A total of nine response categories capture usual intake, ranging from ‘never or less than once/month’ to ‘≥6 times/day.’ The reproducibility and validity of these FFQs have been extensively evaluated^(16–19)^. Nutrient intakes are estimated on the basis of a daily weight assigned to each food item based on its frequency of consumption^(19)^. A database of the nutrient content of FFQ food items maintained by study dietitians, the Harvard nutrient database, began in 1984^(15)^. It has been updated every 4 years to reflect changes to nutrient composition (e.g., changes in *trans* fat content). It also includes new food items and the most recent information on food components based on the U.S. Department of Agriculture (USDA) Nutrient Database for Standard Reference and the Food and Nutrients Database for Dietary Studies.

### Classification of food items into the NOVA groups

The NOVA classification considers the extent and purpose of processing of the food item and includes four groups – (1) unprocessed or minimally processed food, (2) processed culinary ingredients, (3) processed foods and (4) ultra-processed foods. The first three NOVA groups include food products that have undergone processing methods like grinding, roasting, pasteurisation, freezing, vacuum packaging or non-alcoholic fermentation (minimally processed foods), centrifuging, refining or extracting (processed culinary ingredients) or preservation methods such as canning and bottling (processed foods)^(1)^. The category of ultra-processed foods includes food items that normally undergo more intensive industrial processing like hydrolysis, or hydrogenation, extrusion, moulding and pre-frying.

A four-stage process was undertaken to identify the ultra-processed foods from both the adult and the youth FFQs. First, all food items in the FFQs across different waves of data collection were complied. Food items that were nearly identical between FFQs but were presented with minor differences were captured as separate items (e.g., ‘Cold breakfast cereal (1 bowl)’ and ‘Cold breakfast cereal (1 serving)’). This was done to make sure that no food item was overlooked. FFQs from every 4 years of the NHS-I (1986–2010), the NHS-II (1991–2015), the HPFS (1986–2014), from 1996, 1998, 2001 for GUTS-I and from 2004, 2006, 2008, 2011 for GUTS-II were used.

Second, three researchers working independently assigned foods in the adult (N.K, S.R, E.M) and the youth (N.K, M.D, E.M) cohorts to one of the four NOVA groups based on their grade of processing – unprocessed/minimally processed foods (G1), processed culinary ingredients (G2), processed foods (G3) and ultra-processed foods (G4). Food assignment was guided by the definition, examples and supplementary material published by the proponents of the NOVA classification^(1)^. Categorisation was an iterative process requiring the review of the original FFQs used at each wave of data collection to contextualise food items within the larger food lists. Food preparations made from multiple ingredients or different food items that were presented jointly in the FFQ were not disaggregated into their different components. Additionally, the nutrient profile of food items, their actual amounts consumed by the study participants or participant demographics were not considered at any point in the categorisation process. Instead, the original food item as it was listed in the FFQ was categorised in its entirety.

At the third stage, categorisation between researchers was triangulated. Food items for which there was consensus in the categorisation among all researchers were assigned to their NOVA group. A food item was flagged for further scrutiny and shortlisted in case a researcher was unable to assign it to a NOVA group or in cases of disagreement in categorisation by any two researchers.

At stage four, an expert panel comprising of three senior nutrition epidemiologists (F.F.Z; T.F; Q.S) with substantial experience working with the dietary intake in these cohorts, was convened to review and discuss the categorisation of the short-listed products. All discussions were additionally informed by the following resources:

1. Consultations with the research dietitians. The team of research dietitians, led by L.S, was responsible for overseeing the collection of dietary data and for ascertaining the nutrient composition of food items across all Harvard cohorts. They shared their insights obtained from gathering supplementary data, tracking new and reformulated products available in the supermarket, and conducting multiple pilot studies with cohort participants.
2. Cohort-specific documents. These resources provided more insight into the extent of processing of certain FFQ food items by highlighting information on the specific ingredients used in recipes and food preparations, the proportion by weight of individual ingredients to the final recipe or a more detailed description of food items (whether the food was canned or salted or boiled, the brand name of certain packaged foods, etc.).
3. Supermarket scans. The ingredient lists of the first five brands of specific products that were displayed on the Walmart website in 2019 and 2020 were scrutinised. They served as a proxy for establishing the level of processing for a small proportion of food items for which limited information was available from the resources listed above.

The process of categorisation of food items at this stage was also iterative and at the end of stage four, all products were categorised into one of the four NOVA groups. The compilation and categorisation of food items from both the adult and the youth cohorts was done in Microsoft Excel (Microsoft 365, academic license).

## Results

At stage one, a total of 205 unique foods from all FFQ food lists of the NHS, NHS-II and HPFS cohorts and 315 foods in the GUTS cohort were identified and compiled. These included individual food items (‘Butter’; ‘Coffee’; ‘Prunes’; ‘White rice’) and food items that were presented jointly (examples mentioned later). Ninety-seven percent of the food items in the adult cohorts (*n* 199 of 205) and 93 % in the GUTS cohort (*n* 293 of 315) were asked in this manner. Of the multiple food items presented jointly, a large majority of them had similar grades of processing. Examples include ‘Tangerines, clementines, mandarin oranges’, ‘English muffins, bagels, rolls’, ’Shrimp, lobster, or scallops as a main dish’, ‘Beef, pork hotdog’. Some multiple items included specific examples of products or brand names like ‘Hot breakfast cereal, like oatmeal, grits’, ‘Non-fat iced coffee dairy drinks, like Coffee Coolatta, Frappuccino’, ‘Cereal/Granola bar like Nature Valley, Quaker, or Special K’. However, about 3 % (*n* 6 of 205) of the grouped food items in the NHS and the HPFS cohorts and 7 % (*n* 22 of 315) in the youth cohort included a combination of food items with potentially different grades of processing. For instance, in ‘Jams, jellies, preserves, honey’, honey would be differently processed from the other condiments. Similarly, for ‘Pie, home-baked or ready-made’, home-made pies would be differently processed to ready-made pies. Other examples of jointly presented food items with different grades of processing include ‘Onion rings, cooked onions, or soup’, ‘Tofu, soyburgers, miso, edamame, or other soy dish’.

Food items were assigned to a NOVA group by three researchers in stage two. At this stage, other food items included in the FFQs specific to each cohort were also used to inform categorisation. For instance, the classification of ‘Cold breakfast cereal’ into G4 was informed by contextualising it relative to another item on the FFQ, ‘Cooked oatmeal, oatbran’ (G1), for which information was also collected in the same year. Cold cereals were assumed to be packaged and ready-to-eat, and therefore more likely to be ultra-processed, especially as another item was capturing minimally processed oats. For food preparations containing more than one ingredient, the item description helped ascertain the level of processing of certain foods and their categorisation. Examples include ‘Home-made soup without bouillon cube’ (G1), ‘Pie, home-baked’ (G1). The descriptor ‘home-made’ indicated that the food item was likely to not be ultra-processed (confirmed by the phrase ‘without bouillon cube’) and hence it was categorised as G1.

**Fig. 1.**
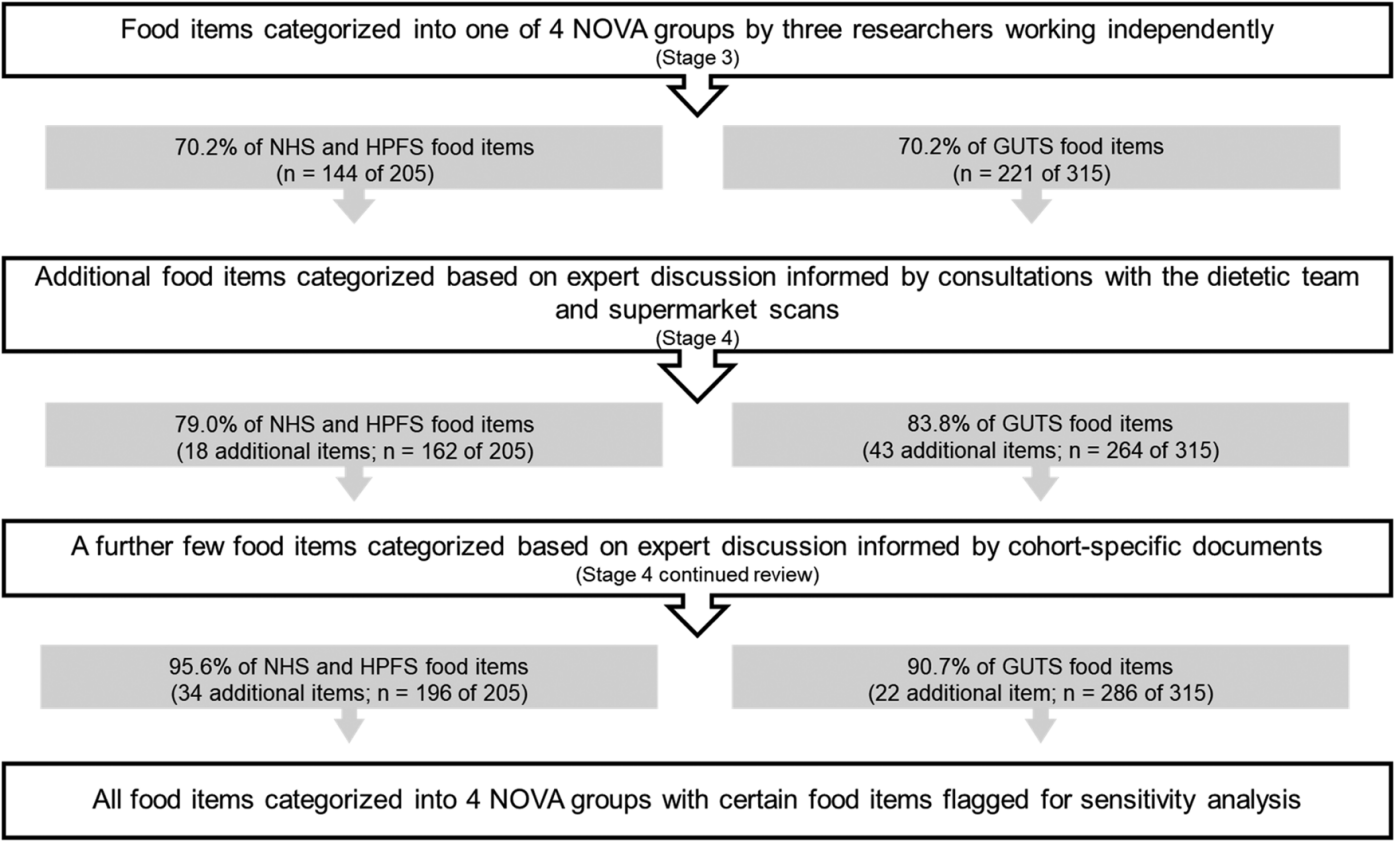
The process of NOVA categorisation of food items captured by semi-quantitative food frequency questionnaires of the Nurses’ Health Studies I and II (NHS), the Health Professionals follow-up study (HPFS) and the Growing Up Today Study (GUTS).

NOVA group assignments were triangulated in stage 3. There was consensus among all study researchers in the categorisation of 144 of the 205 food items (70⋅2 %) in the adult cohorts and 221 of the 315 food items (70⋅2 %) in the youth cohort (see Fig. 1). For example: ‘Rice’, ‘Celery’, ‘Raw carrot’ were assigned to G1; ‘Butter’, ‘Canola oil’ to G2; ‘Canned tuna’, ‘Olives’ to G3; and ‘Jello’, ‘Ready-made soup from a can’ to G4. The descriptor ‘ready-made’ indicated that the food item was likely to be ultra-processed. There was some uncertainty between researchers in the categorisation of 61 of the 205 food items in the adult cohorts (29⋅8 %) and 94 of 315 food items in the youth cohort (29⋅8 %). These included all the grouped foods with a combination of products with potentially different grades of processing mentioned earlier (e.g., ‘Jams, jellies, preserves, honey’).

The food items with uncertain categorisation (*n* 61 in NHS/HPFS; *n* 94 in GUTS) were further reviewed by the expert panel in stage four. Consultations with the dietetic team followed by supermarket scans and team discussions informed the categorisation of eighteen of the sixty-one food items in the adult cohorts and forty-three of the ninety-four food items in the GUTS cohorts. These were subsequently assigned to a NOVA group. At this juncture, 79 % and 83⋅8 % of the food items from the adult and youth cohorts respectively, had been categorised. Sixteen of the eighteen foods in the NHS and HPFS cohorts and twenty-five of the forty-three in the GUTS cohort were identified as having a low potential for contributing to the ultra-processed proportion of the diet – in all possible scenarios of categorisation, these products would have been assigned to either the minimally processed or the processed groups. Examples include: ‘Apricots’, ‘Prunes’, ‘Walnuts’ (G1); ‘Sauerkraut’, ‘Cottage or ricotta cheese’, ‘Mustard’ (G3). The processing category for ‘Mustard’ (G3) was informed by the supermarket scans. Food products (*n* 2 in NHS/HPFS; *n* 18 in GUTS) that were classified as ultra-processed at this stage included ‘Salad dressing’, ‘Soy or Worcestershire sauce’, ‘Tofu, soyburgers, other meat substitutes’, ‘Hawaiian Punch, lemonade, Koolaid or other non-carbonated fruit drinks’ *and food preparations like* ‘Salami, bologna, or other deli meat sandwich’, ‘Bagels, English muffins, or rolls including breakfast sandwiches’, ‘Mixed other dishes (e.g., Pad Thai, chili, Frz. dinners): Beef, pork, or lamb’.

In continued review at stage four, cohort-specific documents along with further input from the dietetic team were used to determine the level of processing for thirty-four additional food items in the adult cohorts and twenty-two additional food items in the GUTS cohort. These products were subsequently assigned to a NOVA category, resulting in the categorisation of 95⋅6 % (adult cohorts) and 90⋅7 % (youth cohort) of all food items. For instance, cohort-specific documents described ‘Applesauce’ as ‘applesauce, canned, sweetened, and without salt’ and ‘Canned peaches’ as ‘peaches, canned, heavy syrup; peaches, canned in juice’ which helped with their assignment into G4. The documents also provided a detailed description for items like ‘French fried potatoes’ (frozen French fries prepared, McDonald's French fries; Burger King French fries). Additional examples of food items that were assigned to the ultra-processed food group at this stage include food preparations like ‘Brownies’, ‘French fried potatoes’, ‘Pizza’, ‘Chowder or cream soup’, ‘Dairy coffee drink’, ‘Danish, sweet rolls, pastry’.

There was not enough evidence in the resource documents to support the classification of the nine remaining food items in the NHS and HPFS cohorts and twenty-nine remaining food items in the GUTS cohort. After discussion with the expert panel, a conservative approach to their categorisation was adopted by assigning these products to a non-ultra-processed NOVA group as their primary categorisation. Examples of the food items that were categorised in this manner include: ‘Popcorn’ (G3); ‘Soya milk’ (G1); ‘Chicken or turkey sandwich’ (G1); ‘Pancakes or waffles’ (G1); ‘Pie, home-baked or ready-made’ (G1). In the future analysis that assesses associations between ultra-processed food consumption and disease outcomes, these products would be recommended for further sensitivity analysis at which point they would be assigned to the ultra-processed group to check for robustness of the associations seen. A flow chart of the categorisation process and cumulative categorised percent is presented in Fig. 1.

**Fig. 2.**
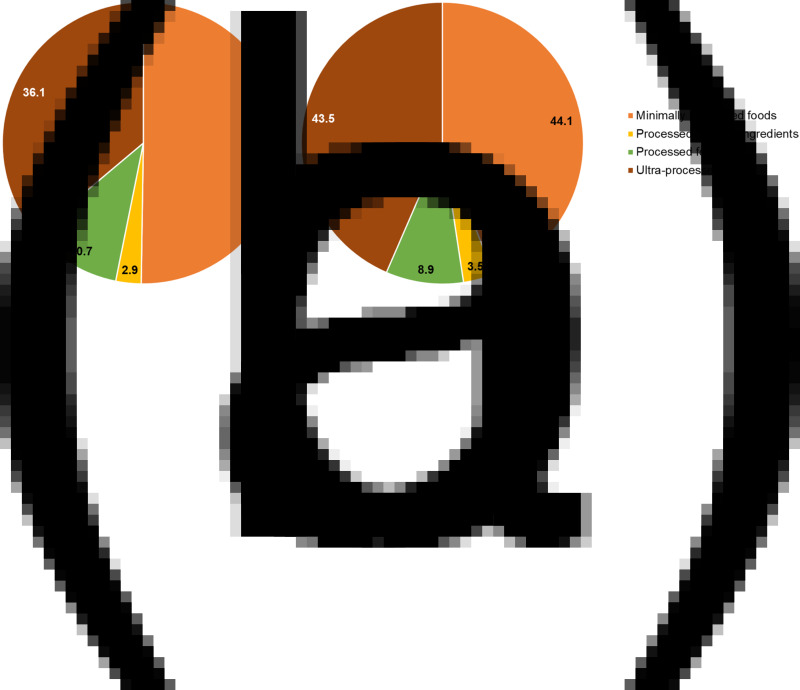
The percentage contribution of the four NOVA groups to the food items compiled from all waves of the Nurses’ Health Studies, the Health Professionals Follow-up Study (a), and the Growing Up Today Studies (b).

A total of 74 of the 205 food items (36⋅1 %) from the NHS and HPFS FFQ food lists and 137 of the 315 food items (43⋅5 %) from the GUTS FFQ food lists were assigned to the ultra-processed food category at the end of the categorisation process at stage 4. Of these, 85⋅1 % of the food items in the adult cohorts (63 of 74) and 72⋅9 % in the youth cohort (100 of 137) were categorised at the end of stage 2, even before discussion with experts (e.g., ‘Regular carbonated beverage with caffeine & sugar’, ‘White bread, pita bread, or toast’, ‘Popsicles’). Over 86 % of the ultra-processed foods from all cohorts were categorised after the first round of reviews with experts. Fig. 2 presents the contribution of the four NOVA groups to the FFQ food lists compiled from all waves of the cohorts.

Tables 1 and 2 capture the short-listed food items scrutinised at stage four.

**Table 1.** Foods items that required discussion and further review in the Nurses’ Health Studies I and II, and the Health Professionals Follow-up Study

All foods with discordant categorisation *n* 61 of 205	Foods requiring additional review *n* 43	Foods in sensitivity analysis *n* 9
Apple juice cider	Applesauce	Beef, pork, lamb sandwich
Applesauce	Beans or lentils	Chicken or turkey sandwich
Apricots	Beef, pork, lamb sandwich	Cream
Beans or lentils	Brownies	Pancakes or waffles
Beef, pork, lamb sandwich	Cake, home-baked	Pie, home-baked or ready-made
Brownies	Canned peaches	Popcorn: regular
Cake, home-baked	Canned pear	Potato chips or corn chips
Canned peaches	Chicken or turkey sandwich	Soya milk
Canned pear	Chowder or cream soup	Tomato sauce
Carrots	Cookies, home-baked	
Chicken or turkey sandwich	Cottage ricotta cheese	
Chowder or cream soup	Cream	
Cooked carrots	Crispbreads	
Cookies, home-baked	Dairy coffee drink	
Maize	French fried potatoes	
Cottage ricotta cheese	Grapefruit juice	
Cream	Grapefruit or grapefruit juice	
Crispbreads	Grapefruit, grapefruit juice	
Dairy coffee drink	Hamburger, lean or extra lean	
French fried potatoes	Home-made sweet roll, coffee cake	
Grapefruit juice	Jams, jellies, preserves, honey	
Grapefruit or grapefruit juice	Orange juice	
Grapefruit, grapefruit juice	Orange juice with calcium	
Hamburger, lean or extra lean	Other canned fruit	
Herbal or decaf tea	Other cheese	
Home-made sweetroll, coffee cake	Other cooked breakfast cereal	
Jams, jellies, preserves, honey	Other fruit juices	
Mustard	Pancakes or waffles	
Orange juice	Pasta	
Orange juice with calcium	Peanut butter	
Other canned fruit	Pie, home-baked or ready-made	
Other cheese	Pizza	
Other cooked breakfast cereal	Popcorn: regular	
Other fruit juices	Potato chips or corn chips	
Other nuts	Potatoes, baked, boiled or mashed	
Pancakes or waffles	Pretzels	
Pasta	Regular hamburger	
Peanut butter	Sour cream	
Peanuts	soya milk	
Peas or lima beans	Tea	
Pie, home-baked or ready-made	Tofu or soyabeans	
Pizza	Tomato sauce	
Popcorn: regular	Tortillas	
Potato chips or corn chips		
Potatoes, baked, boiled or mashed		
Pretzels		
Prunes		
Regular hamburger		
Salad dressing		
Sauerkraut		
Sour cream		
Soya milk		
Tea		
Tofu or soyabeans		
Tomato sauce		
Tortillas		
Walnuts		
Yogurt		
Yogurt-plain, low-carb		
Plain or carbonated water		
Soya or Worcestershire sauce

**Table 2.** Foods items that required discussion and further review in the Growing Up Today Study

All foods with discordant categorisation *n* 94 of 315	Foods requiring additional review *n* 51	Foods in sensitivity analysis *n* 29
Apple juice and other fruit juices	Apples or applesauce	Apples or applesauce
Apples or applesauce	Baked chips, e.g., baked lays	Baked chips, e.g., baked lays
Bagels, English muffins or rolls include breakfast sandwiches	Brownies	Cake
Baked chips, e.g., baked lays	Burrito: beans	Chicken or turkey sandwich
Beans or lentils include baked beans	Burrito: beef or pork	Clear soup (with rice, noodles, vegetables)
Beef (steak, roast) or lamb as main dish	Burrito: chicken or turkey	Corn chips/Doritos
Brownies	Burrito: tofu	Corn chips/Doritos
Burrito: beans	Burrito: vegetables	Cornbread
Burrito: beef or pork	Cake	Cornbread
Burrito: chicken or turkey	Cheeseburger	French toast
Burrito: tofu	Chicken or turkey sandwich	French toast
Burrito: vegetables	Clear soup (with rice, noodles, vegetables)	Grilled cheese
Cake	Coleslaw	Lasagne/baked ziti/ravioli
Cheese	Cookies	Pancakes or waffles
Cheeseburger	Corn chips/Doritos	Pancakes or waffles or French toast
Chicken or turkey as main dish	Corn chips/Doritos	Pancakes or waffles
Chicken or turkey sandwich	Cornbread	Pie
Clear soup (with rice, noodles, vegetables)	Cornbread	Popcorn
Coffee – not decaf	Cream (milk) soups or chowder	Potato chips
Coleslaw	Danish, sweet rolls, pastry	Pudding
Cookies	Doughnuts	Regular potato chips, corn chips, Doritos
Corn chips/Doritos	French fries	Roast beef or ham sandwich
Corn chips/Doritos	French toast	Sandwich or wrap: beef
Cornbread	French toast	Sandwich or wrap: chicken or turkey
Cornbread	Grilled cheese	Sandwich or wrap: tuna
Cottage or ricotta cheese	Hamburger	Sandwich or wrap: veggie (no meat)
Cream (milk) soups or chowder	Lasagne/baked ziti/ravioli	Tea – hot or iced
Danish, sweetrolls, pastry	Macaroni and cheese	Tomato sauce, e.g., spaghetti sauce
Donuts	Pancakes or waffles	Tuna sandwich
Eggrolls	Pancakes or waffles or French toast	
Fish sticks, fish cakes or fish sandwich	Pancakes or waffles	
French fries	Peanut butter sandwich (plain or with jelly, fluff, etc.)	
French toast	Pie	
French toast	Pizza	
Fruit drinks/pouch, lemonade, Sunny D, Koolaid, sugared ice tea or other non-carbonated fruit drink – NOT juice	Popcorn	
Fruit drinks/punch, lemonade, Sunny D, Koolaid or other non-carbonated fruit drink (No juice)	Potato chips	
Grilled cheese	Pudding	
Hamburger	Regular potato chips, corn chips, Doritos	
Hawaiian Punch, lemonade, Koolaid or other non-carbonated fruit drink	Roast beef or ham sandwich	
Hawaiian Punch, lemonade, sport & fruit drinks	Sandwich or wrap: beef	
Jams, jellies, preserves, syrup, honey or fluff exclude sandwiches	Sandwich or wrap: chicken or turkey	
Lasagne/baked ziti/ravioli	Sandwich or wrap: tuna	
Liver	Sandwich or wrap: veggie (no meat)	
Macaroni and cheese	Spaghetti with tomato sauce	
Mixed other dishes (e.g., Pad Thai, chili, Frz. dinners): beef, pork or lamb	Tacos/burritos/enchiladas – beans	
Mixed other dishes (e.g., Pad Thai, chili, Frz. dinners): chicken or turkey	Tacos/burritos/enchiladas – beef	
Mixed other dishes (e.g., Pad Thai, chili, Frz. dinners): fish	Tacos/burritos/enchiladas – beef and beans	
Mixed other dishes (e.g., Pad Thai, chili, Frz. dinners): tofu	Tacos/burritos/enchiladas – chicken	
Mixed other dishes (e.g., Pad Thai, chili, Frz. dinners): vegetables	Tea – hot or iced	
Noodles, pasta	Tomato sauce, e.g., spaghetti sauce	
Onion rings, cooked onions or soup (1/2 cup)	Tuna sandwich	
Orange juice		
Pancakes or waffles		
Pancakes or waffles or French toast		
Pancakes or waffles		
Peaches, plums, apricots (fresh, canned or dried)		
Peanut butter sandwich (plain or with jelly, fluff, etc.)		
Peanuts, nuts		
Peas or lima beans (fresh, frozen, canned) or soup		
Pie		
Pizza		
Popcorn		
Pork, ribs or ham as main dish		
Potato chips		
Potato salad		
Pretzels		
Pudding		
Regular potato chips, corn chips, Doritos		
Rice		
Roast beef or ham sandwich		
Salad dressing (not low energy)		
Salami, bologna or other deli meat sandwich		
Salsa		
Sandwich or wrap: beef		
Sandwich or wrap: chicken or turkey		
Sandwich or wrap: tuna		
Sandwich or wrap: veggie (no meat)		
Shrimp, lobster, scallops		
Soya Milk (glass or with cereal)		
Spaghetti with tomato sauce		
Tacos/burritos/enchiladas – beans		
Tacos/burritos/enchiladas – beef		
Tacos/burritos/enchiladas – beef and beans		
Tacos/burritos/enchiladas – chicken		
Tea – hot or iced		
Tofu		
Tofu, soyaburgers, miso, edamame or other soya dish		
Tofu/soyaburgers/other meat substitutes		
Tomato sauce, e.g., spaghetti sauce		
Tomatoes/tomato juice		
Tortilla – no filling		
Tuna sandwich		
Wine or wine cooler		
Yogurt – not frozen		

The final NOVA classification of food items in the NHS and the HPFS cohorts is presented in Table 3. Table 4 presents the NOVA classification of the GUTS food items. The classification of the food lists of the FFQs formed the basis for the development of four different indicators that reflected participant consumption of ultra-processed foods in the cohorts – absolute kilocalories from ultra-processed foods, percentage of kilocalories from ultra-processed foods, percentage of grams of ultra-processed foods and the servings per day from ultra-processed foods.

**Table 3.** The classification of all food items captured by the semi-quantitative food frequency questionnaires of the Nurses’ Health Studies I and II, and the Health Professionals follow-up study, into the NOVA groups of minimally processed foods (1), processed culinary ingredients (2), processed foods (3), and ultra-processed foods (4)

Food item	NOVA group assignment
1−2 % Milk	1
Alfafa sprouts	1
Apple juice cider	3
Applesauce	4
Apricots	1
Avocado	1
Bacon	4
Banana	1
Beans or lentils	1
Beef, calf, pork liver	1
Beef, lamb as a main dish	1
Beef, pork hot dogs	4
*Beef, pork, lamb sandwich*^a^	1^a^
Beer	3
Beets	1
Blueberries	1
Breaded fish cakes, pieces, sticks	4
Breakfast bar	4
Broccoli	1
Brown rice	1
Brownies	4
Brussels sprouts	1
Butter	2
Caffeine-free coke pepsi	4
Cake, home-baked	1
Cake, ready-made	4
Candy bar with chocolate	4
Candy bar without chocolate	4
Canned peaches	4
Canned pear	4
Canned tuna	3
Cantaloupe	1
Carrots	1
Cauliflower	1
Celery	1
Chicken or turkey hot dogs	4
Chicken or turkey liver	1
*Chicken or turkey sandwich*^a^	1^a^
Chicken or turkey, with skin	1
Chicken or turkey, without skin	1
Chocolate bars	4
Chowder or cream soup	4
Coffee	1
Coke, Pepsi cola	4
Cold breakfast cereal	4
Cooked cabbage	1
Cooked carrots	1
Cooked oatmeal, oatbran	1
Cookies, brownie ready-made	4
Cookies, fat free, reduced fat	4
Cookies, home-baked	1
Maize	1
Cottage ricotta cheese	3
*Cream*^a^	2^a^
Cream cheese	4
Crispbreads	3
Cucumber	1
Dairy coffee drink	4
Dark chocolate bars	4
Dark meat fish	1
Decaffeinated coffee	1
Diet nutrition drinks, Slimfast	4
Doughnuts	4
Dried cranberries	1
Eggplant, zucchini, summer squash	1
Energy bar	4
Energy or high protein bars	4
English muffins, bagels, rolls	4
Ensure, boost or other meal replacement drinks	4
Fat free popcorn	4
Fat free, light crackers	4
Figs	1
Flavoured yogurt without Nutrasweet	4
French fried potatoes	4
Fresh apples, pears	1
Fresh pears	1
Frozen yogurt, sherbet, ice cream	4
Garlic	1
Grapefruit	1
Grapefruit juice	1
Grapefruit or grapefruit juice	1
Green, red peppers	1
Hamburger, lean or extra lean	1
Hawaiian punch	4
Herbal or decaf tea	1
High-protein, low-carb candy bar	4
Home-made soup with bouillon	4
Home-made soup without bouillon cube	1
Home-made sweetroll, coffee cake	1
Hummus	3
Ice cream	4
Iceberg or head lettuce	1
Jams, jellies, preserves, honey	4
Kale, mustard or chard green	1
Ketchup or red chili sauce	4
Light beer	3
Liquor	4
Low-energy soda, caffeine free	4
Low-energy soda, Pepsi, 7-up	4
Low-fat, fat-free mayonnaise	4
Margarine	4
Mixed dried fruit	1
Mixed dried fruits	1
Mixed vegetables	1
Muffins or biscuits	4
Mushrooms	1
Mustard	3
Non-dairy whitener	4
Nutrasweet or Equal	4
Oat bran	1
Oil and vinegar dressing	2
Olive oil added to food	2
Olives	3
Omega-3 fortified including yolk	1
Onion as a garnish	1
Onion as a vegetable	1
Orange juice	1
Orange juice with calcium	1
Orange squash	1
Oranges	1
Other artificial sweetener	4
Other bran	1
Other canned fruit	3
Other carbon beverage	4
Other cheese	3
Other cooked breakfast cereal	1
Other fish	1
Other fruit juices	1
Other grains	1
Other low energy carb	4
Other low energy cola with caffeine	4
Other nuts	1
*Pancakes or waffles*^a^	1^a^
Pasta	1
Peach, apricots, plums	1
Peanut butter	3
Peanuts	1
Peas or lima beans	1
Pepper	1
Pie, home-baked	1
*Pie, home-baked or ready-made*^a^	1^a^
Pie, ready-made	4
Pizza	4
Plain or carbonated water	1
*Popcorn: regular*^a^	3^a^
Pork as a main dish	1
*Potato chips or corn chips*^a^	3^a^
Potatoes, baked, boiled or mashed	1
Pretzels	3
Processed meats, sausage	4
Prune juice	1
Prunes	1
Raisins or grapes	1
Raw carrots	1
Ready-made soup from can	4
Ready-made sweetroll, coffeecake	4
Red chili sauce	4
Red wine	3
Regular crackers	4
Regular hamburger	1
Regular mayonnaise	4
Romaine or leaf lettuce	1
Rye, pumpernickel bread	4
Salad dressing	4
Salami, bologna, processed meat sandwiches	4
Salsa, picante, taco sauce	4
Salt	2
Sauerkraut	3
Shrimp lobster scallop	1
Skim or low-fat milk	1
Snack bars	4
Sour cream	2
*Soya milk*^a^	1^a^
Soya or Worcestershire sauce	4
Spinach, cooked	1
Spinach, raw	1
Splena	4
Spreadable butter	4
Strawberries	1
String beans	1
Sweetroll fat free or reduced fat	4
Tangerines, clementines, mandarin oranges	1
Tea	1
Toasted breads, bagel or English muffin	4
Tofu or soyabeans	3
Tomato juice	3
*Tomato sauce*^a^	3^a^
Tomato soup	4
Tomatoes	1
Tortillas	3
Uncooked cabbage, coleslaw	1
Walnuts	1
Watermelon	1
Wheat germ	1
White bread	4
White rice	1
White wine	3
Whole eggs	1
Whole milk	1
Whole wheat – whole grain bread	4
Yams or sweet potatoes	1
Yogurt	1
Yogurt artificially sweetened	4
Yogurt flavoured without nutrasweet	4
Yogurt-plain, low-carb	1

a Indicates foods to be categorised as ultra-processed (4) for sensitivity analysis.

**Table 4.** The classification of all food items captured by the semi-quantitative food frequency questionnaires of the Growing Up Today Study, into the NOVA groups of minimally processed foods (1), processed culinary ingredients (2), processed foods (3) and ultra-processed foods (4)

Food item	NOVA group assignment
1 or 2 % milk	1
1 % milk	1
2 % milk	1
Added sugar	2
Apple juice, other 100 % fruit juices	1
Apple juice and other 100 % fruit juices	1
Apple juice and other fruit juices	1
*Apples or applesauce*	1^a^
Apples or pears	1
Applesauce	4
Bacon	4
Bacon	4
Bagels, English muffins or rolls include breakfast sandwiches	4
*Baked chips, e.g., Baked Lays*	3^a^
Bananas	1
Beans or lentils including baked beans	1
Beans/lentils/edamame	1
Beans/lentils/soybeans	1
Beef (steak, roast) or lamb as main dish	1
Beef or lamb as a main dish, e.g., as a steak or roast	1
Beef or pork hot dogs	4
Beef, pork or lamb fat	1
Beef/pork sausage	4
Beer	3
Beer, regular	3
Beets (not greens)	1
Biscuit	4
Biscuit/roll	4
Breakfast bars, e.g., Nutrigrain, granola, Kashi	4
Broccoli	1
Broccoli	1
Brown gravy	4
Brown rice	1
Brownies	4
Brussels sprouts	1
Burrito: beans	4
Burrito: beef or pork	4
Burrito: chicken or turkey	4
Burrito: tofu	4
Burrito: vegetables	4
Butter	2
Butter – not margarine	2
*Cake*	1^a^
Cake or snack cakes, e.g., Twinkies	4
Canned tuna	3
Canola oil	2
Cantaloupe	1
Cantaloupe, melons	1
Carbonated, low-cal ‘diet’ beverage with caffeine (e.g., Diet Coke)	4
Carrots, cooked	1
Carrots, cooked	1
Carrots, raw	1
Carrots, raw	1
Celery	1
Celery	1
Cereal/Granola bar, like Nature Valley, Quaker or Special K	4
Cheese	3
Cheese, eaten alone or added to main dish, sandwich or quesadilla	3
Cheeseburger	4
Chicken nuggets	4
Chicken or turkey as a main dish (e.g., fried or roasted) with skin, including grounded	1
Chicken or turkey as a main dish, without skin	1
Chicken or turkey as main dish	1
Chicken or turkey hot dogs or sausages	4
*Chicken or turkey sandwich*	1^a^
Chicken or turkey skin	1
Chocolate like Hershey's or M & M's	4
Chocolate candy like Hershey's, Snickers or M & M's	4
Chocolate candy like Snickers or M & M's	4
Chocolate milk	4
Chocolate milk or any flavoured milk	4
Chocolate or other flavoured milk	4
Chocolate, e.g., Hershey's or M&M's	4
*Clear soup (with rice, noodles, vegetables)*	3^a^
Coffee – not decaf.	1
Coffee drinks – latte, Coolers, Coolatas, Frappuccinos, Mochachinos	4
Cold breakfast cereal	4
Cold breakfast cereal	4
Coleslaw	4
Collard greens/kale	1
Collard greens/kale/cooked spinach	1
Cooked oatmeal, including instant	1
Cookies	4
Maize	1
Maize	1
*Corn chips/Doritos*	3^a^
Corn oil	2
*Cornbread*	3^a^
Cornbread	3
Cottage cheese	3
Cottage or ricotta cheese	3
Crackers, e.g., Cheez-its or Ritz	4
Crackers, like Wheat Thins or Ritz	4
Cream (milk) soups or chowder	1
Cream cheese	4
Cream cheese	4
Cream, e.g., coffee, sour (exclude fat free)	2
Danish, donut, sweetroll or pastry	4
Danish, sweetrolls, pastry	4
Dark bread	4
Dark meat fish, e.g., tuna steak, salmon, sardines, swordfish	1
Decaffeinated coffee	1
Diet soda	4
Donuts	4
Eggrolls	1
Eggs	1
Eggs, e.g., scrambled, fried, in breakfast sandwich	1
Energy bar (like Power or Cliff Bar)	4
Energy bar, e.g., Powerbar, Clif Bar, Lunabar	4
Energy drink – (e.g., Red Bull), Regular energy drinks	4
Energy drink – (e.g., Red Bull), Sugar free, low energy or low carb energy drinks	4
Energy drink – Red Bull, Rock Star	4
English muffins or bagels	4
Fish sticks, fish cakes or fish sandwich	4
French fries	4
French fries	4
*French toast*	1^a^
Fresh fish as main dish	1
Frozen yogurt	4
Frozen yogurt or low-fat ice cream	4
Fruit drinks/pouch, lemonade, Sunny D, Koolaid, sugared ice tea or other non-carbonated fruit drink – NOT juice	4
Fruit snacks or fruit rollups	4
Fun fruit or fruit rollups	4
Graham crackers	4
Grapes	1
Green/red peppers	1
Green/red/yellow peppers	1
Greens/kale	1
*Grilled cheese*	3^a^
Hamburger	4
Hawaiian Punch, lemonade, Koolaid or other non-carbonated fruit drink	4
Hawaiian Punch, lemonade, sport & fruit drinks	4
High protein bar (like MetRx or Balance Bar)	4
High protein bar, e.g., Zone or Balance Bar	4
High protein shake or drink, e.g., whey or soya	4
Hot breakfast cereal, like oatmeal, grits	1
Hot dogs	4
Hot tea	1
Ice cream	4
Ice cream	4
Iced tea – sweetened	4
Instant Breakfast	4
Instant breakfast drink	4
Instant Breakfast Drink/High Protein Shake or Drink	4
Jams, jellies, preserves, syrup, honey or fluff, exclude sandwiches	4
Jello	4
Ketchup	4
Ketchup	4
*Lasagne/baked ziti/ravioli*	1^a^
Lettuce/tossed salad	1
Lettuce/tossed salad	1
Light beer, e.g., Bud Light or Natural Light	3
Liquor, like vodka or rum	4
Liver	1
Low energy or low-fat salad dressing	3
Low energy or low-fat salad dressing	3
Low-fat or whole milk coffee dairy drinks, e.g., Cappuccino, Mocha, Latte	4
Low-fat or whole milk iced coffee dairy drinks, e.g., Coffee Coolatta, Frappuccino	4
Macaroni and cheese	1
Margarine – not butter	4
Mayonnaise	4
Mayonnaise	4
Meatballs or meatloaf	1
Milk	1
Milkshake or frappe	4
Mixed other dishes (e.g., Pad Thai, chili, Frz. dinners): beef, pork or lamb	4
Mixed other dishes (e.g., Pad Thai, chili, Frz. dinners): chicken or turkey	4
Mixed other dishes (e.g., Pad Thai, chili, Frz. dinners): fish	4
Mixed other dishes (e.g., Pad Thai, chili, Frz. dinners): tofu	4
Mixed other dishes (e.g., Pad Thai, chili, Frz. dinners): vegetables	4
Mixed vegetables	1
Mixed vegetables	1
Muffin	4
Nachos with cheese	4
Non-fat coffee dairy drinks, e.g., Cappuccino, Mocha, Latte	4
Non-fat iced coffee dairy drinks, e.g., Coffee Coolatta, Frappuccino	4
Noodles, pasta	1
Oatmeal	1
Oatmeal and other hot breakfast cereal, like farina, grits	1
Olive Oil	2
Onion rings, cooked onions or soup	1
Onions as a garnish or in salad	1
Orange juice	1
Orange juice	1
Oranges, grapefruit	1
Other candy bars (Milky Way, Snickers)	4
Other candy bars, e.g., Milky Way, Snickers	4
Other candy without chocolate (Skittles)	4
Other carbonated, low-cal beverage without caffeine, (e.g., Diet 7-Up)	4
Other cooked breakfast cereal	1
Other fish, e.g., cod, haddock, halibut	1
Other grains, like kasha, couscous, bulgur	1
Other hot breakfast cereal, like farina or grits	1
Other nuts	1
Other regular carbonated beverage with sugar (e.g., 7-Up)	4
*Pancakes or waffles*	1^a^
*Pancakes or waffles or French toast*	1^a^
Pasta (e.g., spaghetti with sauce, lasagne): chicken or turkey	1
Pasta (e.g., spaghetti with sauce, lasagne): vegetable	1
Pasta (e.g., spaghetti with sauce, lasagne): beef/hamburger/pork	1
Pasta (e.g., spaghetti with sauce, lasagne): plain	1
Peaches, plums, apricots	1
Peaches, plums, apricots (fresh, canned or dried)	1
Peanut butter sandwich (plain or with jelly, fluff, etc.)	4
Peanut butter, plain exclude sandwiches	1
Peanuts	3
Peanuts, nut	3
Pears	1
Peas or lima beans	1
Peas or lima beans or soup	1
*Pie*	1^a^
Pizza	4
*Popcorn*	3^a^
Popsicles	4
Poptarts	4
Pork, ribs or ham as main dish	1
Posicles	4
*Potato chips*	3^a^
Potato salad	1
Potato salad	1
Potatoes – baked, boiled, mashed	1
Potatoes – baked or boiled, mashed	1
Pretzels	3
*Pudding*	1^a^
Pudding – EXCLUDE sugar free	4
Raisins	1
Regular carbonated beverage with caffeine & sugar, (e.g., Coke, Pepsi, Mt. Dew, Dr. Pepper)	4
Regular hot tea with caffeine, including green tea	1
*Regular potato chips, corn chips, Doritos*	3^a^
Rice	1
*Roast beef or ham sandwich*	1^a^
Safflower oil	2
Salad dressing (not low energy)	1
Salad dressing (not low energy)	1
Salami, bologna or other deli meat sandwich	4
Salsa	1
Salsa	1
*Sandwich or wrap: beef*	1^a^
*Sandwich or wrap: chicken or turkey*	1^a^
Sandwich or wrap: peanut butter & jelly/fluff	4
Sandwich or wrap: salami, bologna or other deli meat	4
*Sandwich or wrap: tuna*	1^a^
*Sandwich or wrap: veggie (no meat)*	1^a^
Sausage	4
Seeds (Sunflower or Pumpkin)	1
Seeds, e.g., sunflower or pumpkin	1
Shrimp, lobster or scallops as a main dish	1
Shrimp, lobster, scallops	1
Skim/non-fat milk	1
Skim/non-fat milk (glass or with cereak)	1
Smoothies (e.g., medium Jamba Juice or Orange Julius)	1
Snack cakes, like Ring Dings/Swiss Rolls/Twinkies	4
Snack cakes, like Twinkies	4
Soda – not diet	4
Soda – not diet	4
Soya milk (glass or with cereal)	4
Soya milk, any flavour	4
Soyabean oil	2
Spaghetti or other pasta with tomato sauce	1
Spaghetti with tomato sauce	1
Spinach	1
Spinach, raw as in salad	1
Sport drinks – Powerade or Gatorade	4
Spreadable butter (butter mixed with oil to make it soft and spreadable)	4
Strawberries	1
String beans	1
String beans	1
Tacos/burritos/enchiladas	4
Tacos/burritos/enchiladas – Beans	4
Tacos/burritos/enchiladas – Beef	4
Tacos/burritos/enchiladas – Beef and Beans	4
Tacos/burritos/enchiladas – Chicken	4
*Tea* – *hot or iced*	1^a^
Tofu	3
Tofu, soyaburgers, miso, edamame or other soya dish	4
Tofu/soyaburgers/other meat substitute	4
Tomato juice or V8	1
*Tomato sauce, e.g., spaghetti sauce*	3^a^
Tomatoes	1
Tomatoes	1
Tomatoes/tomato juice	1
Tortilla – no filling	3
Tortilla, e.g., tacos, quesadillas (exclude burritos)	3
*Tuna sandwich*	3^a^
Vegetable oil	2
Veggieburger	4
Walnuts	1
Water – tapped or bottled	1
Watermelon	1
Wheat or dark bread	4
Whipped cream	4
Whipped cream – EXCLUDE coffee drinks and/or fat free	2
White bread, pita bread or toast	4
White or pita bread, exclude sandwiches	4
White rice	1
Whole milk	1
Whole milk (glass or with cereal)	1
Whole wheat or whole grain bread, include toast	4
Whole wheat or whole grain, exclude sandwiches	4
Wine or wine cooler	4
Yams/sweet potatoes	1
Yams/sweet potatoes	1
Yogurt – not frozen	4
Yogurt – plain, not frozen	1
Yogurt – artificially sweetened (e.g., light peach)	4
Yogurt – sweetened (e.g., strawberry, vanilla)	4
Zucchini, summer squash, eggplant	1

Items will not add up to 315 – only one of two or more near-identical items (e.g., Corn chips/Doritos; Corn chips or Doritos) have been presented here.

a Indicates foods to be categorised as ultra-processed (4) for sensitivity analysis.

## Discussion

This article details the process of categorising food items into the four NOVA groups and identifying the ultra-processed proportion of the FFQ food lists of the NHS-I and II, the HPFS and the GUTS cohorts. Over 70 % of the food items across all cohorts were assigned into one of the four NOVA groups after the first attempt at categorisation, based on published definitions that account for differences in processing between groups and their accompanying example products. The approach to classifying the remaining 30 % of food items involved discussions with experts that were informed by insights from the research dieticians, information provided by cohort-specific documents and scans of online grocery stores.

A conservative approach to the classification of some of the more challenging food items was adopted. This meant that only food items that could be justifiably considered ultra-processed based on information from cohort-specific documents were assigned to this NOVA group. The nutrient composition of the product was never considered in the categorisation process. In the handful of instances of uncertainty, a non-ultra-processed NOVA group was assigned as the primary classification and the food items were flagged for future sensitivity analysis where they could be re-categorised as ultra-processed. It is therefore reasonable to assume that the ultra-processed foods may be underestimated, and caution is needed in the interpretation of the future absolute intake of ultra-processed foods estimated from these FFQs. While this may have an attenuating effect on any future diet–disease relationship studied, the recategorisation planned for sensitivity analyses will help measure the variability associated with this approach.

Besides the resources mentioned in this article to help with the NOVA categorisation of dietary data, additional resources may be considered. Since there is no one gold-standard for applying the NOVA categorisation, the use of year-appropriate, context- or region-specific nationally representative surveys that use 24-h diet recalls or diet records could be used to gather more detailed information on dietary intake. The food items from these surveys could help determine the level of processing of certain challenging products. For instance, the National Health and Nutrition Examination Surveys (NHANES) in the US could be used to provide some insight into the processing grade of certain challenging foods if there was no access to cohort-specific resources. Information on complementary aspects of participant dietary behaviour like their frequency of eating out, purchase of branded packaged foods, perceived consumption of branded products, etc., may also be used to corroborate the NOVA classification of their diet. Previously, researchers have used participant report of percentage of brands of ultra-processed foods and home-made foods to resolve uncertainty and reach consensus in categorising dietary data collected using 24-h diet records^(20)^. This additional information was used after all consumed food items had been categorized according to NOVA by study dietitians and reviewed by an expert panel.

Alternative approaches were considered but not implemented. In the present approach, composite food preparations with multiple ingredients were considered in their entirety when being assigned to a NOVA group, informed by key information contained in their item descriptions (home-made, ready-made, takeaway, frozen, etc.), along with information of the ingredient lists of equivalent products from the websites of leading retailers. Alternatively, home-made composite dishes could be divided into their ingredients and the individual ingredients could then be assigned to a NOVA group. Another alternative approach might classify food items into more than one NOVA group with appropriate resources justifying the allocation of a percentage of the food item into each group. For instance, ‘Pie, home-baked or ready-made’ could be split to allocate 60 % of its nutrient profile to the minimally processed group and 40 % to the ultra-processed group. This approach of dividing the nutrient profile of the food item into more than one NOVA group has been done before^(6,21)^. It may either be an equal split between different NOVA groups or be divided in a proportion that is informed by other sources^(21)^. Validation studies will be needed to estimate the misclassification minimised in determining the ultra-processed foods by these approaches.

Once validated, this approach could be used in future studies to minimise the misclassification associated with estimating the ultra-processed fraction of the diet and could help assess diet–disease relationships more accurately. It could also be used to identify ultra-processed foods from databases that contain information on dietary intake, food product acquisition, or food item sales. The identification of specific barriers faced at each stage of the categorisation process could be used to inform data-driven algorithms categorising dietary intake and inform the refinement of existing dietary assessment instruments to more accurately reflect the level of processing of food items. Including specific information in dietary assessment instruments on the processing of certain food items (e.g., ‘home-made from scratch’), including brand names of packaged products where possible (for branded breakfast cereal and breads for instance), capturing the place of preparation (at restaurant, street-food, take-aways), as well as the manner of preparation of mixed dishes and the types of ingredients used (e.g., ‘from scratch with fresh ingredients’, ‘pre-made and frozen using processed ingredients’) would make identifying the level of processing of food items easier^(22)^. For FFQs, it could also mean adding more items or sub-dividing existing ones to differentiate between grades of processing and asking follow-up questions that give a better sense of the overall processing of the dietary pattern^(23)^.

Finally, this work may serve as a protocol for applying the NOVA classification and identifying ultra-processed foods in other large-scale cohort studies. While it presents a specific example of an approach for the classification of FFQ food item, the various stages and the decision-points detailed in this manuscript could be modified based on context-specific needs and applied to other studies. This work may also be valuable as a template for authors thinking about documenting and making transparent their method of classification to help increase the reproducibility of their research.

### Strengths and limitations

The limitations of this approach are important to highlight. First, the present approach to classification assumed no changes to the food composition of the food item over time. As a result, the nutrient database from 2014 was used to determine the nutrient composition for all products irrespective of whether that product was listed in the FFQ in 1986 (first FFQ cycle in these cohorts) or in 2015 (the latest available FFQ cycle in these cohorts). The use of the nutrient database from 2014 likely confers a healthier nutrient composition to products that have been subject to reformulation^(24,25)^. Second, changes to the grade of processing over time because reformulation of the products was not captured by the present approach. Thus, the classification of a food product as ultra-processed remained static over time. While it is likely that a small portion of foods switched between NOVA groups over the course of dietary data collection due to reformulation, future work would be needed to capture the evolution of the processing of these products.

A third limitation is related to the use of supermarket scans in informing categorisation. The asynchrony between the collection of the dietary information from participants and the website searches may result in the identification of different brands of products from the ones consumed by the participants and/or different levels of processing as gauged from the ingredient lists. In the present approach, ingredient lists of the most popular brands of products from 2019 to 2020 were used to reflect the processing of certain food items listed in FFQs from 1986 to 2015. To minimise the potential for misclassification, cohort-specific resources were given priority in informing the NOVA categorisation of most food items and the grocery website scans, while helpful, were only used to categorise a handful of products.

Finally, the food lists included a few food items that combined individual foods from different NOVA groups. While it is likely that the energetic contribution of each food was small, the approach to categorisation did not attempt to disaggregate the grouped foods to estimate the dietary contribution of each of the individual items. Single food items that were composite dishes (like baked dishes requiring multiple ingredients, some of which may be ultra-processed) were also not disaggregated into individual components, but instead, the dish in its entirety was categorised into a NOVA group.

The strengths of this approach were the triangulation of NOVA group assignment, expert review of the categorisation process, the use of supporting documents to inform the categorisation of the more challenging food items and the high transferability of the approach to categorising dietary data collected using other diet assessment methods in different contexts. The actual consumption levels as reported by the participants or participant demographics were not considered, so inherent systematic biases associated with the over- under-reporting of certain foods did not influence the categorisation strategy adopted.

## Conclusions

This manuscript presents the strategy used in the identification of the ultra-processed portion of the food lists of FFQs in large-scale population studies. The iterative, conservative approach adopted, relied on discussions with experts and was informed by insights from the research dieticians, information provided by cohort-specific documents and scans of online grocery stores. All food items were assigned a primary NOVA group with some foods being ear-marked for further sensitivity analysis. Future work would be needed to certify the validity of this approach by comparing participant ultra-processed food consumption estimated through FFQs against diet records while using the present approach of dietary categorisation. An evaluation of the generalisability and feasibility of applying this approach to other study populations and contexts is also warranted. Documentation and discussions of alternative approaches for categorisation and the evolution of dietary assessment methods to better capture ultra-processed foods are encouraged.

## Availability of data and material

The datasets used and/or analyzed during the current study are available from the corresponding author on reasonable request.
